# Camel milk and fermented camel milk prevent dextran sulfate sodium-induced ulcerative colitis via the intestinal flora-short-chain fatty acids-mucosal barrier axis in mice

**DOI:** 10.3389/fmicb.2025.1723833

**Published:** 2025-12-16

**Authors:** Weisheng Xu, Qigeqi Dong, Qingwen Guo, Ruihua Li, Manzar Abbas, Ling Li, Chunlin Zhang, Guofen Zhao

**Affiliations:** 1 Key Lab of Germplasm Innovation and Utilization of Triticeae Crops at Universities of Inner Mongolia Autonomous Region, College of Life Sciences, Inner Mongolia Agricultural University, Hohhot, China; 2 Inner Mongolia Key Laboratory of Biomanufacturing Technology, College of Life Sciences, Inner Mongolia Agricultural University, Hohhot, China; 3 Inner Mongolia Saikexing Institute of Breeding and Reproductive Biotechnology in Domestic Animal, Hohhot, China; 4 Kalaqin Banner Wangyefu Town Sishijiazi Health Center, Chifeng, China

**Keywords:** fermented camel milk, gut microbiota, intestinal barrier function, mouse model, preemptive intervention, SCFAs, ulcerative colitis

## Abstract

**Introduction:**

Ulcerative colitis poses a significant threat to human health. Dysbiosis of the gut microbiota and decreased levels of short-chain fatty acids are known contributors to the pathogenesis of ulcerative colitis. While camel milk and fermented camel milk have demonstrated beneficial effects in alleviating intestinal inflammation, the differences in their preventive efficacy against ulcerative colitis and the underlying mechanisms remain unclear. Dextran sulfate sodium can induce ulcerative colitis, at least in part, through activation of the NF-κB/MAPK signaling pathway. This study aims to investigate the differential preventive effects of camel milk and fermented camel milk against dextran sulfate sodium-induced ulcerative colitis in mice and to elucidate the underlying mechanisms mediated by the gut microbiota and short-chain fatty acids.

**Results:**

Through the study, we found that preemptive intervention with camel milk and fermented camel milk significantly mitigated dextran sulfate sodium-triggered pathological manifestations, including body weight loss (e.g., from 97%in DSS group to 99% in FTC group, *p* < 0.01), elevated disease activity index (from a peak score of 2.8 to 2.0, *p* < 0.01), colonic shortening (from 5.8 cm to 6.2 cm, *p* < 0.01). Both treatments also restored intestinal barrier integrity, increasing the expression of tight junction proteins, and elevated the anti-inflammatory cytokineIL-10 (*p* < 0.05). Then, it can also regulate the intestinal flora imbalance in mice with ulcerative colitis. Finally, we found that both can also reduce the reduction of short-chain fatty acids in mice with ulcerative colitis (*p* < 0.05). Notably, the fermented camel milk exhibited superior efficacy compared to the camel milk in mitigating fermented camel milk-induced body weight loss, the decline of IL-10and E-cadherin levels, microbial diversity decreasing, the Bacteroidetes/Firmicutesratio increasing, and the relative abundance of *Lachnospiraceae* NK4A136 group decreasing in ulcerative colitis mice.

**Discussion:**

Our findings demonstrate that preemptive intake of camel milk and fermented camel milk effectively prevents ulcerative colitis by preserving the intestinal barrier, regulating immune responses, and restoring gut microbiota homeostasis and short chain fatty acids production, with fermented camel milk offering enhanced benefits. We hold optimistic prospects for camel milk and fermented camel milk as dietary supplements to prevent ulcerative colitis and stabilize gut microbiota homeostasis.

## Highlights

- At the same time, the effects of preventive intake of camel milk and fermented camel milk on chemical-induced ulcerative colitis were compared.
- It connects gut microbiota dysbiosis to reduced protective short-chain fatty acids, ultimately leading to intestinal barrier damage and inflammatory activation.

## 1 Introduction

Ulcerative colitis (UC) is a non-specific chronic relapsing–remitting inflammatory bowel disease (IBD) (Kadayat et al., 2017), has exhibited a significant rise in global prevalence in recent decades, and is classified by the World Health Organization (WHO) as a “modern refractory disease” (Ng et al., 2017; Su et al., 2022; Xu et al., 2022). UC pathogenesis involves persistent damage to the colonic and rectal mucosa and submucosa, manifesting as confluent open ulcers (Lasa et al., 2022). Intestinal mucosal damage leads to increased intestinal permeability, allowing luminal contents and gut microbiota to breach the lamina propria barrier, thereby exacerbating intestinal inflammation (Bray et al., 2018). The gut microbiota dysbiosis, characterized by reduced abundance of *Lactobacillus* and *Bifidobacterium*, impairs intestinal barrier integrity and immune regulatory capacity while promoting pathobiont expansion, thereby exacerbating disease severity (Chen X. Q. et al., 2021). Current clinical management of UC primarily relies on pharmacological interventions and surgical procedures. Pharmacological approaches include aminosalicylates, glucocorticoids, immunosuppressants, and biologics (Barberio et al., 2021; Solitano et al., 2023; Yamada et al., 2019). However, these therapies fail to adequately address persistent and recurrent inflammatory activity (Gao et al., 2025). Surgical intervention, typically involving proctocolectomy, carries a risk of reduced quality of life, increased mortality, and leads to cancer (Ocansey et al., 2020; Singh et al., 2015). Therefore, there is a clear need to develop safer and more effective strategies, particularly those focused on prevention.

Probiotics can restore gut microbiota equilibrium resulted in alleviation of UC symptoms through modifying immunomodulatory effects, metabolite production (e.g., SCFAs), and promotion of intestinal mucosal regeneration. Notably, alterations in microbiota-derived metabolites, particularly short chain fatty acids (SCFAs) and tryptophan derivatives such as indole-3-propionic acid (IPA), are intricately linked to disease progression (Ambat et al., 2024; Gao et al., 2025). The gut microbiota-derived metabolite indole-3-propionic acid (IPA) modulates mucosal CD4^+^ T cell functions, thereby alleviating intestinal mucosal inflammation (Gao et al., 2025). Under varying conditions, SCFAs exhibit a dual nature: they not only stimulate the expansion of IL-10-producing T cells and regulatory Tregs but can also drive the differentiation of naïve T cells into effector T cells (T-bet^+^ IFN-γ^+^ Th1 cells or IL-17^+^ Th17 cells). This differentiation is instead directly mediated by HDACi activity (Parada Venegas et al., 2019). Notably, the amelioration of UC symptoms critically depends on restoring the relative abundance of beneficial taxa (e.g., *Lactobacillus*, *Bifidobacterium*) and suppressing pathogenic bacterial populations (Li et al., 2021; Zhou et al., 2021). A key mucin-degrading bacterium, *Akkermansia muciniphila*, has garnered significant attention for its ability to enhance gut barrier integrity and exert anti-inflammatory effects, thereby conferring protection against metabolic and inflammatory diseases (Derrien et al., 2017). Probiotic strains, including *Lactobacillus reuteri* (Yan et al., 2024), *Lactobacillus plantarum* (Kang et al., 2021; Zhang et al., 2018), *Leuconostoc* spp., and *Prevotella* spp. (Mancabelli et al., 2017), *Bifidobacterium* spp. (Ishikawa et al., 2003), *Clostridium butyricum* (Wu et al., 2022), and *Bifidobacterium pseudolongum* (Guo et al., 2022), have been shown to maintain gut microbiota homeostasis, alleviate clinical symptoms, and suppress intestinal inflammation in UC. However, the beneficial health effects of probiotics may vary depending on strain specificity and formulation characteristics.

As an alternative, naturally functional foods like camel milk (TC) offer a multi-component and historically validated source of bioactive compounds—including lactoferrin, immunoglobulins, and antioxidant peptides—that may support gut health more comprehensively (Alhassani, 2024). Recent studies have demonstrated that TC effectively reduces blood glucose levels and aids in the management of diabetes mellitus. Emerging evidence also supports its exploratory therapeutic applications for autism spectrum disorder (ASD) and certain cancers. It had been demonstrated that TC could exert hypoglycemic and antithrombotic (anticoagulant) effects in streptozotocin (STZ)-induced diabetic rats (Aida et al., 2020). TC fermentation with probiotics yields FTC (fermented camel milk), a probiotic-enriched functional food that not only harbors a high density of viable probiotics but also contains elevated antioxidant components, including oligosaccharides, vitamins, bioactive peptides, and conjugated linoleic acid (CLA) (Fernández et al., 2015; Xu et al., 2023). In the FTC, lactic acid bacteria (LAB) predominate, with *Lactobacillus* spp. constituting 35% of the 93 LAB strains isolated from the FTC (Buchilina and Aryana, 2021). It was demonstrated that carbon tetrachloride (CCl₄)-induced toxicity and aberrant physiological effects could be effectively mitigated through the use of *Lactococcus lactis*-fermented TC, attributed to its potent antioxidant, immunomodulatory, and anti-inflammatory properties (Hamed et al., 2018). Despite these attributes, the preventive potential of TC and FTC against UC, and their different preventive effects and possible synergy with gut microbiota and SCFAs, remain poorly investigated.

Through the previous research in the laboratory, based on the analysis of metabolites of fermented TC by LC-MS, we found that fermented TC contains a large number of bioactive substances related to inflammation, such as creatine and ascorbic acid (Xu et al., 2023). We hypothesized that preemptive intervention with TC and FTC would attenuate dextran sulfate sodium (DSS)-induced colitis in mice by modulating gut microbiota composition, enhancing SCFA production, and restoring intestinal barrier and immune balance. We further hypothesized that FTC would exhibit superior efficacy due to its enhanced bioactive profile. To test these hypotheses, we systematically evaluated the preventive effects of TC and FTC using histopathological, immunological, and metagenomic approaches, aiming to provide mechanistic insights into their protective role and support their potential as dietary strategies for UC prevention.

## 2 Methods and materials

### 2.1 Materials

#### 2.1.1 Mice

Forty female C57BL/6J mice (6-week-old, body weight 16 ± 4 g) under specific pathogen-free (SPF) conditions were purchased from SPF (Beijing) Biotechnology Co., Ltd. [Animal License Number: SCXK(Jing) 2019-0010]. During the experimental period, mice were housed under controlled environmental conditions with a temperature of 25 ± 2 °C, relative humidity of 50 ± 5%, and a 12 h light/dark cycle. All animals were provided ad libitum access to distilled water and a standard chow diet [maintenance feed supplied by SPF (Beijing) Biotechnology Co., Ltd.].

#### 2.1.2 Probiotic strains

The five lactic acid bacterial strains utilized in this study were *Lactobacillus plantarum* strain KC28, *Lactobacillus plantarum* strain K25, *Lactobacillus* sp. D1501, *Leuconostoc mesenteroides* strain SHU1396, and *Lactobacillus acidophilus* strain 2,888, all provided by the Biochemistry and Molecular Biology Laboratory at Inner Mongolia Agricultural University.

#### 2.1.3 Camel milk

TC was collected from herders’ households in the Alxa region, filtered, pasteurized, and stored at −20 °C in a laboratory freezer for subsequent use.

#### 2.1.4 Reagents

DSS (Meilunbio Co., Ltd.), 10% formaldehyde solution (Sinopharm Chemical Reagent Co., Ltd.), 4% paraformaldehyde (Sinopharm Chemical Reagent Co., Ltd.), sodium pentobarbital (Sigma), hematoxylin (Solarbio), eosin (Solarbio), neutral resin (Solarbio), paraffin (Shanghai Sinopharm Group Co., Ltd.), xylene (Shanghai Sinopharm Group Co., Ltd.), goat anti-rabbit IgG-HRP (Shanghai Sinopharm Group Co., Ltd.), and rabbit serum (Solarbio).

### 2.2 Methods

#### 2.2.1 Preparation of fermented camel milk

Five strains of *Lactobacillus plantarum* strain KC28, *Lactobacillus plantarum* strain K25, *Lactobacillus* sp. D1501, *Leuconostoc mesenteroides* strain SHU1396, and *Lactobacillus acidophilus* strain 2,888 were activated in MRS medium (1%, V/V) and cultured at 37 °C and 180 rpm for 12–16 h. Repeat the same steps above to expand culture. The bacterial solution was uniformly mixed at a ratio of 2:1:3:5:3 (V/V) according to the above order. The total volume of the bacterial solution was 24 mL, centrifuged (6,000 rpm, 5 min), discarded supernatant, washed with normal saline, centrifuged again, discarded supernatant, and collected precipitate, the viable count of precipitate was 6.33 × 10^9^ CFU/mL measured by dilution plate method. Twenty milliliters TC was added to the precipitate, fully mixed, and fermented at 38 °C for 24 h. After fermentation, it was stored at 4 °C (Xu et al., 2023). The viable count of fermented TC was 1.45 × 10^10^ CFU/mL measured by dilution plate method. The pH of fermented TC measured by pH meter was 4.35.

#### 2.2.2 Construction of mice model of UC

Prior to the experimental procedures, all mice underwent a one-week acclimatization period with ad libitum access to standard chow and distilled water. Following the acclimatization period, the 40 mice were randomized using GraphPad Prism software (version 9.5.0) according to their initial body weight to ensure that there were no significant differences between groups at the beginning of the experiment. The 40 mice were distributed into following four groups (*n* = 10 per group): CK (control group): no treatment, DSS (DSS-induced model group): received DSS to induce UC, TC (camel milk treatment group): preemptive intervention with sterilized TC, and FTC (fermented camel milk treatment group): preemptive intervention with FTC.

**Figure 1:**
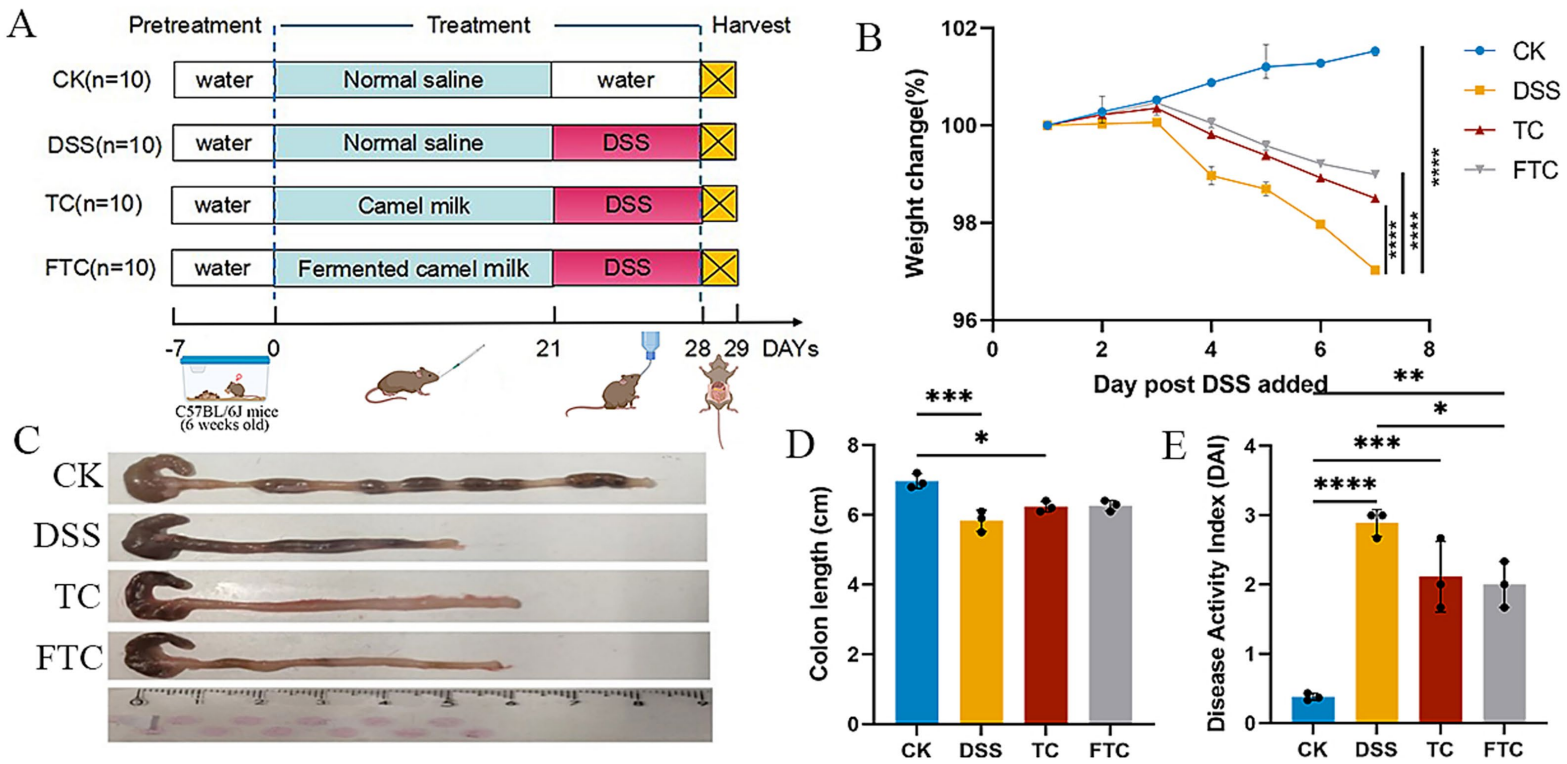
Pretreatment with camel milk (TC) and fermented camel milk (FTC) protects against DSS-induced colitis in mice by preserving body weight and colon integrity. **(A)** Schematic diagram of the experimental design. Mice were pretreated with water, TC, or FTC for 14 days prior to DSS administration (*n* = 10 biologically independent mice per group). **(B)** Changes in body weight following DSS-induced colitis. FTC pretreatment significantly prevented DSS-induced weight loss, indicating superior protective efficacy (*n* = 3). **(C)** Representative macroscopic images of colons from each group. The TC group exhibited the least colon shortening. **(D)** Quantitative analysis of colon length. TC and FTC pretreatment ameliorated DSS-induced colon shortening (*n* = 3). **(E)** Disease activity index (DAI) scores. FTC pretreatment resulted in the lowest disease activity index, consistent with its enhanced protective effect (*n* = 3). In CK, TC, and FTC groups, ^*^p < 0.05, ^**^p < 0.01, and ^***^p < 0.001 vs. DSS group using one-way ANOVA with Tukey’s *post-hoc* test.

Throughout the experimental period (days 0–21), all mice were provided ad libitum access to a standard maintenance diet and distilled water. The CK and DSS groups received daily oral gavage of 5 mL/kg·bw sterile saline, while the TC and FTC groups were administered with 5 mL/kg·bw of sterilized TC (65 °C, 30 min) and FTC, respectively. From day 22 of the experimental period onward, DSS, TC, and FTC groups were provided with 3% (w/v) DSS solution for 7 consecutive days, while the CK group continued receiving distilled water. During this 7-day phase, body weight was measured and recorded daily, and physical condition (e.g., posture, activity) and stool consistency (e.g., diarrhea, occult blood) were closely monitored and documented. The entire experimental duration spanned 28 days. On the final day, all mice were fasted for 12 h before euthanasia by cervical dislocation under sodium pentobarbital anesthesia. Colons were excised, measured for length, and fixed in 4% paraformaldehyde for histopathological analysis. Fecal samples were collected, flash-frozen in liquid nitrogen, and stored at −80 °C for subsequent profiling (Figure 1A). The determination and evaluation of all outcome indicators were performed in a blind state.

#### 2.2.3 Recording of the mice’s body weight and fecal conditions

For the 21 days, the body weight of the mice was measured every 3 days. Their physical condition was observed, and their body weight was recorded. Starting from the 22nd day of the experiment, the body weight of the mice was measured and recorded daily. The body weight change rate of each group of mice during the model-building period was calculated. The fecal occult blood status of mice in each group was detected using the occult blood kit (XF5011, GEMIC, China), and color changes were observed and recorded (Supplementary Table S1). The disease activity index (DAI) was determined by assessing the percentage of body weight loss, stool consistency, and fecal occult blood (or gross bleeding) according to the criteria provided in Table 1.

**Table 1:** DAI score calculation.

Score	Weight loss (%)	Stool consistency	Blood in feces
0	0	Normal	Negative (no bleeding)
1	1–5	/	Weak positive
2	5–10	Loose stools	Positive (slight bleeding)
3	10–15	/	Strong positive
4	>15	Watery diarrhea	Gross bleeding

#### 2.2.4 Histopathological and immunohistochemistry analysis of colon tissue

Colonic tissue samples were paraffin-embedded and sectioned into 4-μm-thick slices. The sections were stained with hematoxylin and eosin (HE). Histological damage in the colon was observed under light microscope, and histopathological scoring was performed on colonic tissues from each group following established criteria (Cooper et al., 1993). Tissue sections were deparaffinized and rehydrated through a graded alcohol series. Incubating the sections in sodium citrate buffer (10 mM, pH 6.0) at 95 °C for 20 min using a water bath. After cooling to room temperature naturally, the sections were rinsed with PBS. Endogenous peroxidase activity was quenched by incubation with 3% hydrogen peroxide, followed by blocking of nonspecific binding sites with 5% normal goat serum. Sections were subsequently incubated overnight at 4 °C with primary antibodies against ZO-1 (ab221547, abcam, 1:500, Rabbit Source), occludin (27260-1-AP, proteintech, 11000, Rabbit Source), and claudin-1 (20874-1-AP, proteintech, 15000, Rabbit Source). After being rinsed three times with PBS, sections were incubated with secondary antibodies (ab6721, abcam, 1:1000, Goat Source) for 1 h at room temperature. Immunoreactivity was visualized using DAB chromogen, with hematoxylin counterstaining applied to visualize nuclei and digital images were captured. Histopathological scoring of immunohistochemical results was performed following the standardized criteria (Olaya et al., 2015).

#### 2.2.5 Determination of inflammatory factors levels

Colon tissue samples were analyzed using ELISA kits to determine the levels of inflammatory cytokines, including TNF-α (JM-02415M2, Jiangsu Jingmei Biotechnology Co., Ltd., China), IFN-γ (JM-02465M2, Jiangsu Jingmei Biotechnology Co., Ltd., China), IL-1β (JM-02323M2, Jiangsu Jingmei Biotechnology Co., Ltd., China), IL-6 (JM-02446M2, Jiangsu Jingmei Biotechnology Co., Ltd., China), IL-4 (JM-02448M2, Jiangsu Jingmei Biotechnology Co., Ltd., China), and IL-10 (JM-02459M2, Jiangsu Jingmei Biotechnology Co., Ltd., China), in mouse colon tissues. All procedures were performed by following manufacturer’s protocol.

#### 2.2.6 qRT-PCR was used to detect the expression level of the tight junction protein

Total RNA was extracted from colon sample using a RNA queous^™^ Microextraction Total RNA Isolation Kit (AM1931, ThermoFisher, United States), and reverse transcription was performed to synthesize cDNA using the Hifair^®^ III 1st Strand cDNA Synthesis SuperMix for qPCR (gDNA digester plus) (Yeasen, HB221101, China). *β-actin* was selected as an internal reference gene for normalization, and the relative gene expression level was calculated using the 2^−ΔΔCT^ method. The primer sequences used in this study were obtained from the official website of the primer library (Table 2). The reaction mixture of qRT-PCR is shown in Supplementary Table S2. Programs: initial denaturation (95 °C 30 s), quantification (95 °C 5 s, Tm 30 s) for a total of 45 cycles, melting (95 °C 5 s, Tm 60 s, 95 °C), and cooling (50 °C 30 s) (Zhang et al., 2023).

**Table 2:** Primer sequences for qRT-PCR.

Gene	Primer sequences (5′ to 3′)
*β-actin*-F	GCTCTGGCTCCTAGCACCAT
*β-actin*-R	GCCACCGATCCACACAGAGT
*E-cadherin*-F	AACCTGAAGGCAGCCGACA
*E-cadherin*-R	GTTGCCCCACTCGTTCAGATAA
*Occludin*-F	CCGTCTAATCAATCTTTGCAGC
*Occludin*-R	AGTTTGGAGAAGTCACCGCAG
*ZO-*1-F	TCACGATCTCCTGACCAACG
*ZO-*1-R	TGACGGGTAAATCCACATCTG

#### 2.2.7 Determination of short-chain fatty acids content

In this experiment, the content of short chain fatty acids was determined by absolute quantitative method. Accurately take acetic acid, propionic acid, butyric acid, isobutyric acid, valeric acid, isovaleric acid standard, according to the volume ratio of 20:10:5:2:2:1 mixing, constant volume to 100 mL, get 6 kinds of acid mixed standard, then diluted with ultrapure water to obtain four different concentration gradient mixed standard solution (1×, 0.5×, 0.25×, 0.01×). When the sample was loaded, 200 μL mixed standard solution, 700 μL ultrapure water, 100 μL 100 μg/mL n-butanol were added, and n-butanol was used as internal standard. The standard curve was obtained with the standard acid concentration as the abscissa and the peak area as the ordinate.

Fresh feces (0.20 g) was homogenized in 1.60 mL of sterile deionized water, followed by vortexing and let stand at room temperature for 20 min. The mixture was then centrifuged at 15,000 rpm for 15 min at 4 °C. The supernatant was transferred to a new EP tube. The fecal pellet was resuspended in another 1.6 mL of sterile deionized water, and the procedure described above was repeated. The resulting supernatants were combined and mixed thoroughly, then filtered through a 0.22 μm membrane filter. The filtered supernatant (200 μL) was mixed with 700 μL of sterile water and 100 μL of 100 μg/mL n-butanol to prepare the final sample (Jiang et al., 2020). Following sample preparation, SCFAs profiling was performed via gas chromatography-flame ionization detector (GC-FID) under optimized chromatographic conditions. SCFAs analysis was executed using a PE Clarus 680 gas chromatograph equipped with an Elite-FFAP capillary column (30 m × 0.25 mm inner diameter × 0.25 μm film thickness). Chromatographic conditions were optimized as follows: injection volume of 1 μL, split ratio of 1:10, detector temperature of 260 °C, injector temperature of 240 °C, and a temperature program for the column oven starting at 120 °C (held for 8 min), increasing at 2 °C/min to 180 °C (final hold time: 2 min). Carrier gas flow rates were set to 45.0 mL/min for hydrogen (H₂), 450.0 mL/min for air, and 1.0 mL/min for nitrogen (N₂).

#### 2.2.8 Gut microbiome analysis

Microbiome analysis of fecal samples were performed by Nanjing Jisi Huiyuan Biotechnology Co., Ltd. Genomic DNA (gDNA) was extracted from mice fecal samples using TIANGEN stool kits (DP328-02, TIANGEN, China), followed by PCR amplification of the V4–V5 hypervariable regions of the bacterial 16S rRNA gene with universal primers 515F (5′-GTGCCAGCMGCCGCGGTAA-3′) and 907R (5′-CCGTCAATTCMTTTRAGTTT-3′). PCR products were purified using the AxyPrep DNA Gel Extraction Kit (AP-GX-50, Axygen, China), and their concentration and quality were assessed using a Qubit^®^ 3.0 Fluorometer (Thermo Fisher Scientific, United States). Purified amplicons were pooled and sequenced on an Illumina MiSeq platform (Illumina, United States). Raw sequencing data were processed using the vsearch software (version 2.15.0) (Rognes et al., 2016), with chimeric sequences removed via a combined *denovo* and reference-based approach (Uchime algorithm). Taxonomic classification was performed against the SILVA database (release 138) on the I-Sanger Cloud Platform,^1^ including operational taxonomic unit (OTU) clustering, alpha/beta diversity analysis, microbial composition profiling, and differential abundance testing. Beta-diversity was assessed based on Bray–Curtis dissimilarity, and statistical significance of group differences was determined using PERMANOVA with 999 permutations.

#### 2.2.9 Correlation analysis

At the genus level, the abundance of the four groups of intestinal flora was compared in pairs to screen out the intestinal flora with significant differences (*p* < 0.05). Then, correlation analysis was performed between colon length, DAI scores, inflammatory factors levels, tight junction protein expression scores, SCFAs concentrations, and gut microbial taxa exhibiting significant differential abundance at the genus level, using cloud-based bioinformatics tools on the OmicStudio Platform.^2^ Finally, the intestinal flora with a significant correlation was retained for correlation analysis.

#### 2.2.10 Statistical analysis

Statistical analysis was performed using GraphPad Prism software (version 9.5.0). Quantitative data are expressed as mean ± standard deviation (mean ± SD). Intergroup differences were analyzed by one-way analysis of variance (ANOVA) followed by Tukey’s multiple range test for *post-hoc* comparisons. A significance threshold of *p* < 0.05 was applied to determine statistically significant differences.

## 3 Result

### 3.1 Preemptive intervention with TC and FTC attenuated pathological manifestations in DSS-induced UC in mice

Throughout the experiment, no abnormal symptoms were observed in the CK group. In contrast, mice in the DSS group exhibited rapid body weight reduction starting from day 4 post-intervention, accompanied by diarrhea and hematochezia (Figure 1B). The DAI score in the DSS group reached a maximum value of 3 (Figure 1E). Although body weight loss was also observed in TC and FTC groups, both the TC and FTC groups showed a significantly lower weight reduction rate compared to the DSS group (both *p* < 0.001). Compared to the DSS group, reduced DAI scores were observed in both TC and FTC groups, with a particularly significant difference demonstrated in the FTC group relative to DSS-treated mice (Figure 1E, *p* < 0.05). Furthermore, experimental data revealed that prophylactic intervention with TC and FTC significantly mitigated colonic shortening in colitis mice (Figures 1C,D).

### 3.2 Preemptive intervention with TC and FTC resisted colonic pathological injury in DSS-induced UC in mice

**Figure 2:**
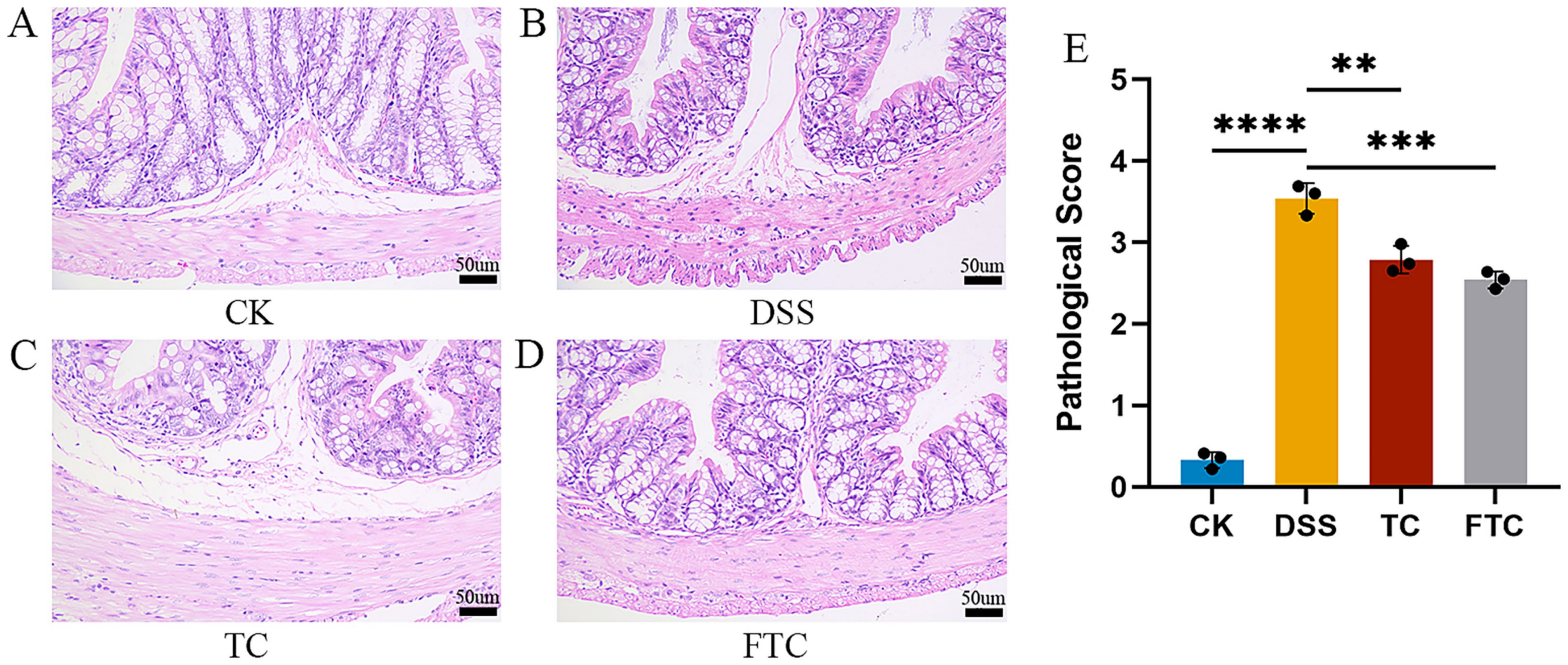
Observation and scoring of colon sections in mice. **(A–D)** H&E tissue sections of mice, images were acquired under a 200× objective lens. **(A)** The CK group (*n* = 3), **(B)** the DSS group (*n* = 3), **(C)** the TC group (*n* = 3), **(D)** the FTC group (*n* = 3). **(E)** Pathological score. FTC pretreatment resulted in lower pathological score (*n* = 3). In CK, TC, and FTC groups, ^*^p < 0.05, ^**^p < 0.01, and ^***^p < 0.001 vs. DSS group using one-way ANOVA with Tukey’s *post-hoc* test.

UC model animals exhibited significant pathological alterations in colonic architecture, while the histopathological scoring system objectively quantified disease progression through systematic evaluation of inflammatory cell infiltration, epithelial integrity, and crypt distortion. To investigate pathological alterations in UC, colonic tissues were subjected to HE staining combined with histopathological scoring. Higher histopathological scores corresponded to more severe tissue damage. Figures 2A–D illustrates histopathological features of colonic tissues across experimental groups. The CK group displayed preserved colonic architecture with orderly arranged mucosal epithelial cells, intact crypt structures, abundant goblet cells in the mucosal layer, and tightly organized submucosal connective tissue, accompanied by negligible inflammatory cell infiltration. In contrast, DSS-treated mice exhibited severe architectural distortion, including focal mucosal epithelial necrosis, marked depletion of goblet cells, and extensive inflammatory cell infiltration within the lamina propria. The TC group and the FTC group presented with mild structural abnormalities, but maintained the integrity of the epithelium. The tissue was characterized by partial loss of goblet cells, with no obvious inflammatory infiltration. There was no significant difference in the staining section results between the two groups. Histopathological evaluation revealed significant intergroup variations in colonic pathology scores (Figure 2E). The CK group demonstrated the lowest histopathological scores, while the DSS group mice exhibited the highest scores (*p* < 0.001). Both TC and FTC groups showed significantly reduced pathology scores compared to the DSS group (*p* < 0.01). However, no significant difference was observed between the TC and FTC groups’ interventions (*p* > 0.05).

### 3.3 Preemptive intervention with TC and FTC alleviates colonic inflammatory responses in DSS-induced UC in mice

**Figure 3:**
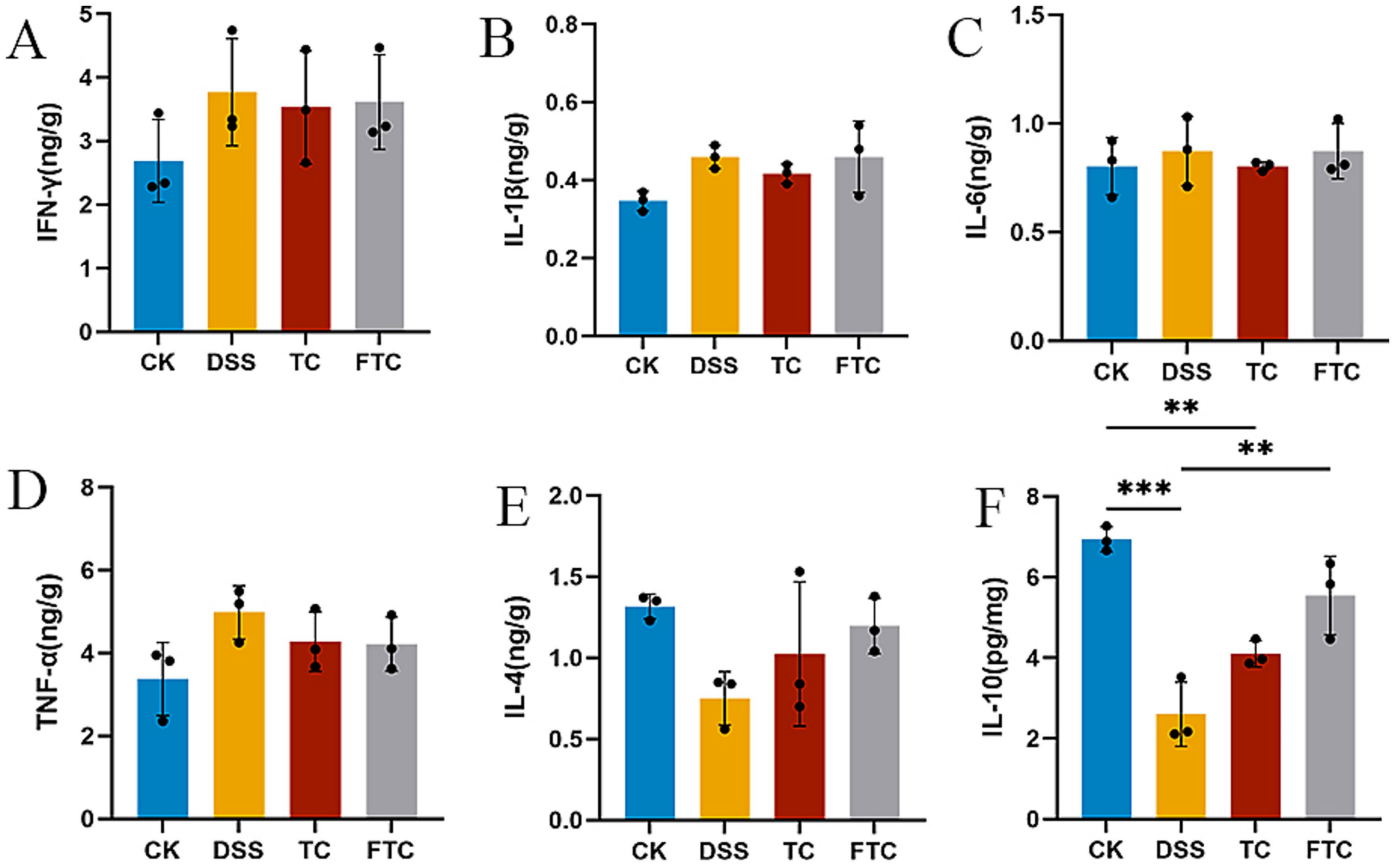
The expression level of inflammatory factors in mice. **(A)** The content of the INF-γ pro-inflammatory factor in mice, **(B)** the content of the IL-1β pro-inflammatory factor in mice, **(C)** the content of the IL-6 pro-inflammatory factor in mice, **(D)** the content of the TNF-α pro-inflammatory factor in mice, **(E)** the content of the IL-4 anti-inflammatory factors in mice, **(F)** the content of the IL-10 anti-inflammatory factors in mice. In CK, TC, and FTC groups, ^*^p < 0.05, ^**^p < 0.01, and ^***^p < 0.001 vs. DSS group using one-way ANOVA with Tukey’s *post-hoc* test.

The pathophysiological progression of UC is intrinsically linked to a self-perpetuating inflammatory cascade. Inflammatory cytokines serve as molecular initiators of this process, while the resultant inflammatory milieu further potentiates cytokine hypersecretion through positive feedback mechanisms. Therefore, we measured and compared the changes of inflammatory cytokines in all four groups (CK, DSS, TC, FTC). Preemptive Intervention with TC and FTC demonstrated immunomodulatory effects in murine colitis models (Figures 3A–F). Both treatments effectively suppressed DSS-induced upregulation of pro-inflammatory cytokines (TNF-α, IFN-γ, IL-1β, and IL-6; vs. DSS group) (Figures 3A–D), while concurrently attenuating the decline of anti-inflammatory cytokines (IL-4 and IL-10; vs. DSS group) (Figures 3E,F). In summary, preemptive intervention with TC and FTC alleviated colonic inflammation in DSS-induced murine colitis by suppressing pro-inflammatory cytokine expression and attenuating the decline of anti-inflammatory cytokines.

### 3.4 Preemptive intervention with TC and FTC preserves colonic barrier integrity in DSS-induced UC in mice

The pathogenesis of UC involves concurrent inflammatory responses and intestinal barrier dysfunction. Tight junction proteins (TJPs), including E-cadherin (mediating epithelial cell adhesion) and occludin (sealing paracellular spaces), constitute critical components of the intestinal barrier. We employed immunohistochemical (IHC) staining with histopathological scoring and qRT-PCR to assess the expression of these barrier-related proteins.

**Figure 4:**
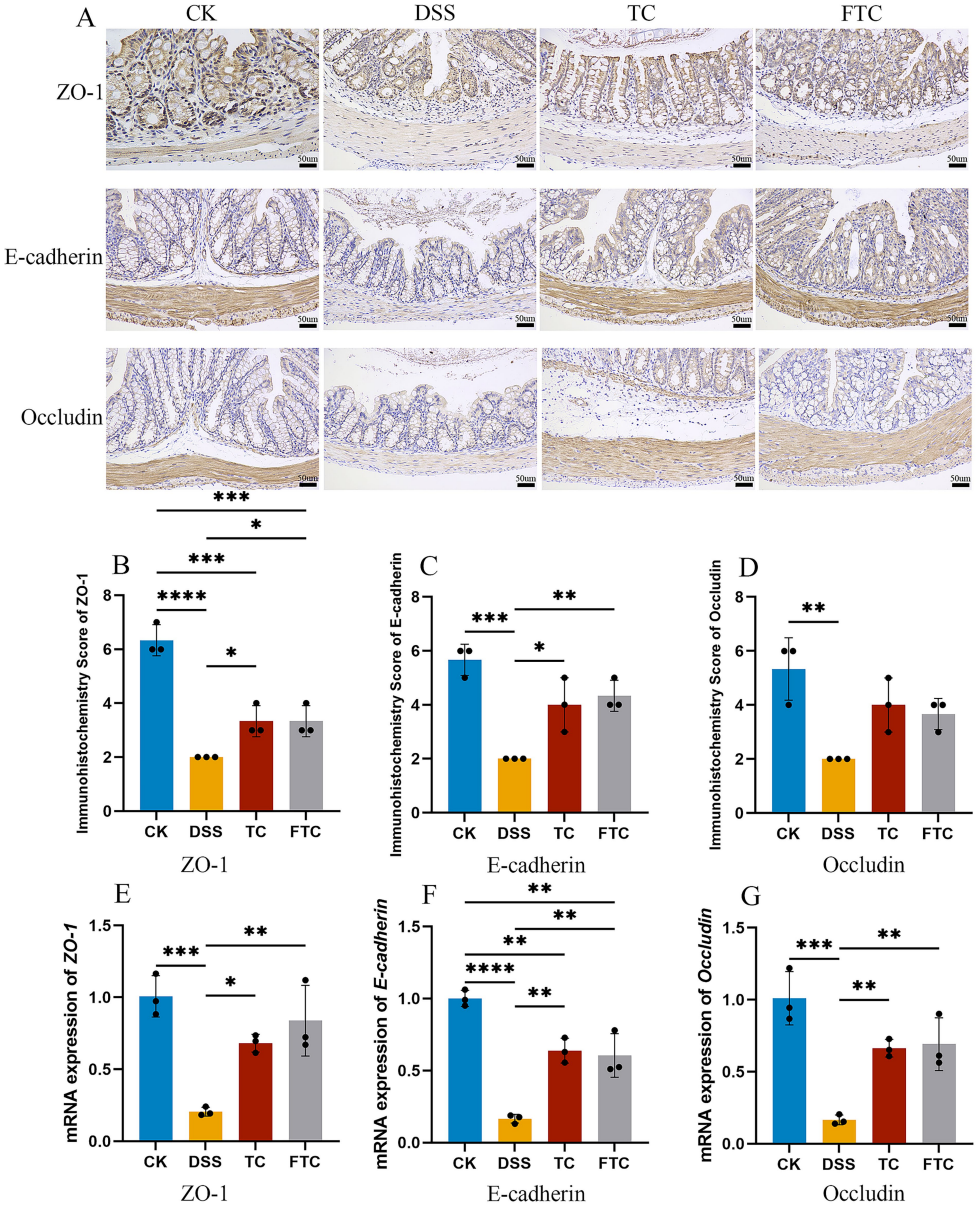
Immunohistochemical results of mouse colon and the relative mRNA expression of three tight junction proteins. **(A)** Immunohistochemical results of three tight junction proteins, images were acquired under a 200× objective lens, **(B–D)** immunohistochemical scores of three tight junction proteins, **(E)** the relative mRNA levels of *ZO-1* gene, **(F)** the relative mRNA levels of *E-cadherin* gene, **(G)** the relative mRNA levels of *Occludin* gene. In CK, TC, and FTC groups, ^*^p < 0.05, ^**^p < 0.01, and ^***^p < 0.001 vs. DSS group using one-way ANOVA with Tukey’s *post-hoc* test.

Immunohistochemistry with scoring demonstrated that ZO-1 (Figures 4A,B), E-cadherin (Figures 4A,C) in TC and FTC groups were largely enhanced in the colon as compared with DSS group, and qRT-PCR assays showed that TC and FTC groups significantly improved the mRNA expressions of ZO-1 (Figure 4E), E-cadherin (Figure 4F), and Occludin (Figure 4G) in the colon. Interestingly, the FTC group protected the expression of E-cadherin and ZO-1 mRNA compared to the TC group.

### 3.5 Preemptive intervention with TC and FTC modulates intestinal SCFAs profiles in DSS-induced UC in mice

**Figure 5:**
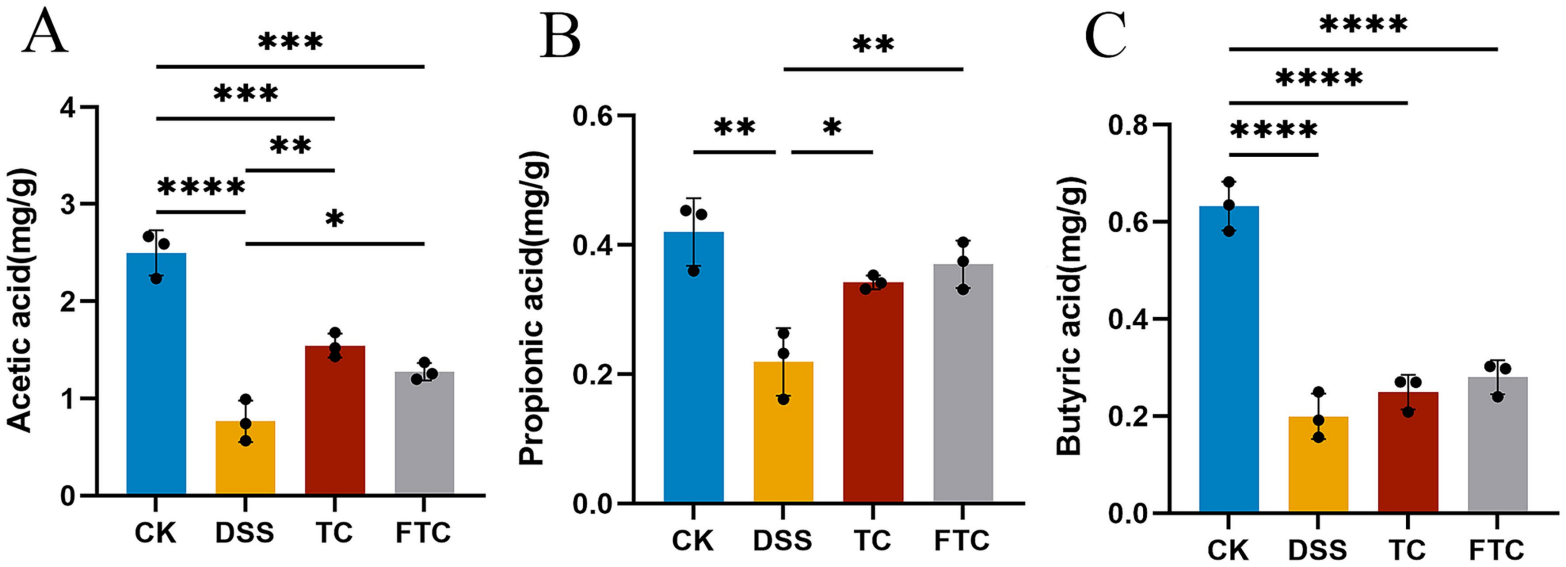
Short-chain fatty acid content in mice. **(A)** The content of acetic acid in mouse feces, **(B)** the content of propionic acid in mouse feces, **(C)** the content of butyric acid in mouse feces. In CK, TC, and FTC groups, ^*^p < 0.05, ^**^p < 0.01, and ^***^p < 0.001 vs. DSS group using one-way ANOVA with Tukey’s *post-hoc* test.

SCFAs, primarily including acetic acid, propionic acid, and butyric acid, regulate intestinal microbial homeostasis, preserve the intestinal epithelial barrier, and exert anti-inflammatory effects, all of which are critical for animal health. GC analysis revealed significant alterations in SCFAs profiles across experimental groups. Compared with CK group, the contents of acetic acid, propionic acid and butyric acid in DSS group decreased significantly (Figures 5A–C, *p* < 0.01). Compared with CK group, the content of acetic acid and butyric acid in TC group and FTC group decreased significantly (Figures 5A,C, *p* < 0.01). Compared with DSS group, the contents of acetic acid and propionic acid in TC group and FTC group were significantly increased (Figures 5A,B *p* < 0.05).

### 3.6 Preemptive intervention with TC and FTC modulates gut microbiota composition in DSS-induced UC in mice

**Figure 6:**
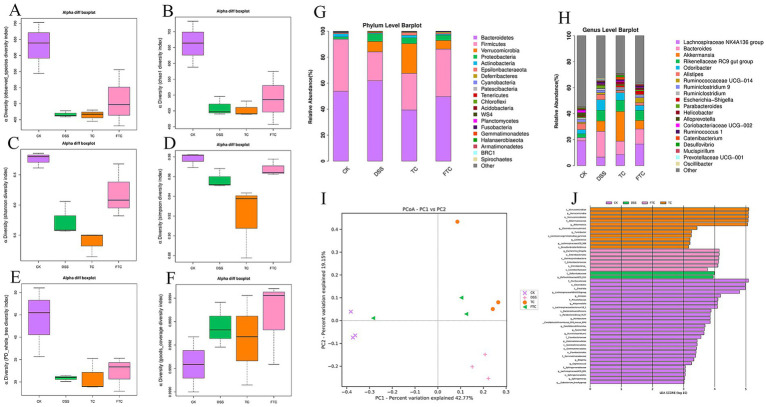
Analysis of the mouse gut microbiome. **(A–F)** α-Diversity analysis. **(G)** Species distribution at the phylum level. **(H)** Species distribution at the genus level. **(I)** β-diversity analysis. **(J)** Significantly different intestinal flora species.

Alpha diversity analysis was conducted using indices including Chao 1, Shannon, and Simpson to evaluate microbial richness in the intestinal microbiota of mice (Figures 6A–F), while β-diversity analysis was performed to investigate differences in microbial community composition among groups, principal coordinates analysis (PCoA) revealed a clear separation of gut microbial communities among the groups (Figure 6I), this separation was statistically significant as confirmed by PERMANOVA (*R*^2^ = 0.682, *p* = 0.001). The result was further supported by ANOSIM (*R*^2^ = 0.715, *p* = 0.001). Preemptive intervention with TC and FTC have made the α-diversity indices closer to CK mice. These results demonstrated that both TC and FTC effectively modulate intestinal microbial dysbiosis in mice with ulcerative colitis (UC).

To elucidate the precise composition of the gut microbiota, taxonomic analysis was conducted at both phylum and genus levels. At the phylum level, Bacteroidetes and Firmicutes were identified as the dominant phyla in murine intestinal microbiota (Figure 6G), collectively accounting for over 94% of total bacterial abundance across all experimental groups. Compared with the CK group, the DSS induced group showed a remarkable increase in the relative abundance of Bacteroidetes, alongside a reduction in the relative abundance of Firmicutes, while an increase in the B/F ratio. Compared with the DSS group, both the TC and FTC groups exhibited an increase in the relative abundance of Firmicutes and a decrease in Bacteroidetes, resulting in a lower Bacteroidetes/Firmicutes (B/F) ratio (Figure 6G). Subsequent taxonomic analysis at the genus level revealed differences in microbial composition among groups (Figure 6H). The three predominant genera in the DSS group were *Bacteroides*, *Odoribacter*, and *Akkermansia*, whereas *Akkermansia*, *Bacteroides*, and *Rikenellaceae* RC9 gut group were predominant in the TC group. Notably, the context-dependent role of *Akkermansia* may be influenced by dietary intervention and mucosal status, potentially contributing to its variation across groups under different physiological conditions. In the FTC group, *Lachnospiraceae* NK4A136 group, Bacteroides, and *Rikenellaceae* RC9 gut group displayed the highest relative abundances. Notably, these genera were predominantly classified under the Firmicutes and Bacteroidetes. To identify bacterial taxa potentially associated with UC pathogenesis, LEfSe (linear discriminant analysis effect size) analysis was conducted to compare intergroup microbial community structures (Figure 6J). *Defluviitaleaceae* and *Defluvitaleaceae* UCG-011 were found to be enriched in the DSS group, which may be potential biomarkers specific to DSS-induced UC.

### 3.7 Gut microbiota correlation analysis with integrated pathophysiological indices in DSS-induced UC in mice

**Figure 7:**
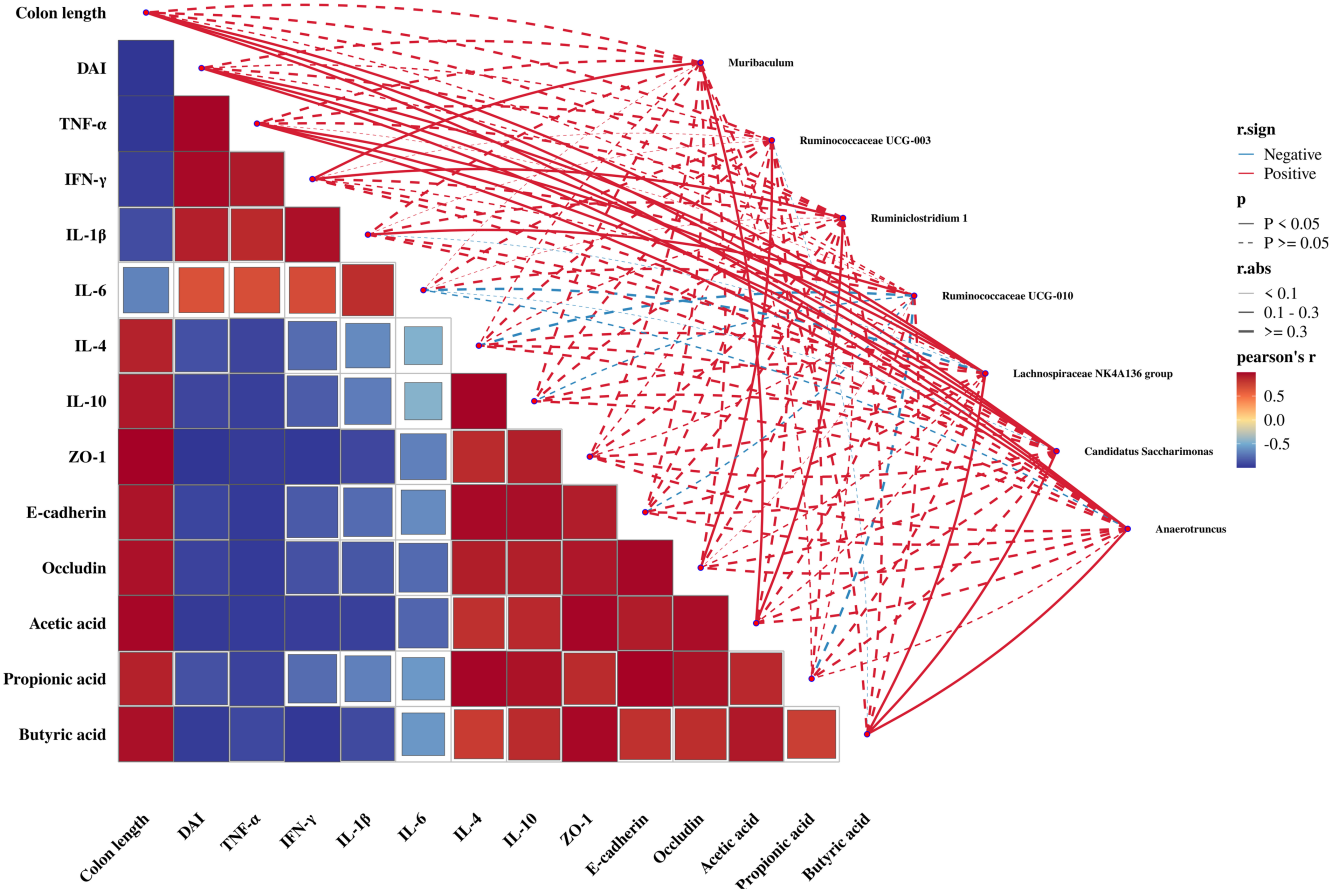
Significantly different intestinal flora and integrated pathophysiological indices correlation network heat map. The relationship between colonic physiological indexes, inflammatory factors, intestinal barrier proteins, short-chain fatty acids, and intestinal flora at genus level was demonstrated. The color (red/blue) of the heat map corresponds to the correlation coefficient (positive/negative correlation); the network graph connection indicates the direction of association, significance (*p* < 0.05 is the solid line), and intensity. Statistical methods: Pearson correlation analysis.

To investigate whether alterations in gut microbiota structure influence inflammatory severity and colonic barrier protection, a comprehensive correlation analysis was conducted between intestinal flora and clinical parameters including colitis features (colon length, DAI), inflammatory cytokines (TNF-α, IL-6, etc.), intestinal barrier integrity, and SCFA content (Figure 7). First of all, a total of 38 intestinal bacteria with significant differences were screened (Supplementary Table S3, *p* < 0.05). Among the 38 intestinal bacteria, a total of 7 bacteria were significantly correlated with the physiological indices. Significant positive correlations were identified between *Lachnospiraceae* NK4A136 group, *Candidatus Saccharimonas*, and *Anaerotruncus* with colon length, DAI scores, TNF-α levels, and butyrate concentrations (*p* < 0.05), while negative correlations were observed with IL-6 expression (*p* > 0.05). These findings point to a complex and contradictory set of relationships. *Muribaculum* and *Ruminococcus* 1 existed in dramatic positive correlation with IFN-γ and acetic acid content; *Ruminococcaceae* UCG-003 had a remarkable positive correlation with occludin content; and a strong positive correlation was demonstrated between *Ruminococcaceae* UCG-010 and IL-1β content. These findings suggest that these intestinal flora may play pivotal roles in mitigating colitis progression through modulation of inflammatory responses and maintenance of mucosal homeostasis.

## 4 Discussion

In recent years, interest in the research on probiotic properties and TC has significantly increased. Compared to bovine milk, TC has been shown to possess a superior nutritional and bioactive profile, characterized by a higher concentration of vitamin C, iron, antimicrobial peptides, and immunoglobulins, along with the absence of β-lactoglobulin, a major milk allergen (He et al., 2022; Ho et al., 2022). With emerging evidence demonstrating TC therapeutic potential in managing diabetes mellitus, thrombosis, and enteritis (Fernández et al., 2015; Xu et al., 2023). Probiotic strains have been shown to ameliorate UC through modulating gut microbiota composition and enhancing immune homeostasis, while promoting intestinal mucosal regeneration. *Lactobacillus plantarum* strain KC28 can significantly reduce the body weight of obese mice induced by intermediate high-fat diet (Huang et al., 2021). *Lactobacillus plantarum* strain K25 may have the probiotic function of lowering cholesterol (Aziz et al., 2024). There are few studies on the four probiotics we used. FTC serves as an optimal probiotic delivery matrix, not only harboring diverse beneficial microbial consortia (e.g., *Lactobacillus* spp., *Bifidobacterium* spp.) but also containing augmented bioactive components that collectively confer enhanced antioxidant, immunomodulatory, and anti-inflammatory capacities beyond those of TC (Hamed et al., 2018). Studies have shown that milk fat globule membrane protein in TC can protect UC by regulating intestinal flora and amino acid metabolism in mice (Kang et al., 2025). TC polar lipids ameliorate DSS-induced colitis in mice by modulating the gut microbiota (He et al., 2024). Our study demonstrated that preemptive intervention with TC and FTC significantly attenuated DSS-induced UC in mice, with FTC exhibiting superior therapeutic efficacy in preserving intestinal barrier integrity and suppressing inflammation compared to TC.

Body weight variation, DAI, and colonic morphology serve as critical parameters for evaluating UC severity. DSS directly targets colonic epithelial cells, inducing mucosal damage characterized by depletion of mucin-secreting goblet cells, epithelial erosion, and ulceration (Korish, 2014). This breach in the colonic epithelial barrier significantly elevates intestinal permeability, facilitating translocation of commensal bacteria and luminal antigens across the compromised mucosal barrier, which subsequently triggers innate immune activation and chemotactic signaling (Arab et al., 2014). Our results demonstrated that preemptive intervention with TC and FTC significantly alleviated UC symptoms, as evidenced by reduced DAI scores, attenuated body weight loss, preserved colon length, and diminished inflammatory cell infiltration. Notably, FTC showed better efficacy in defending against DSS-induced colitis severity (Figures 1B,E, 2E). These findings indicated that both TC and FTC confer prophylactic protection against UC. Prior studies administering 4% DSS solution to mice for 7 consecutive days demonstrated significant colonic shortening versus controls, while dietary interventions restored colon length to near-normal range (Yin et al., 2020), which was consistent with our results. TC administration had been shown to mitigate DSS-induced colonic mucosal damage and immune cell dysregulation, while reducing DAI and histopathological scores (Al-Awadi and Srikumar, 2001). These findings were largely consistent with our experimental results, though discrepancies in the magnitude of therapeutic effects were observed across specific biomarkers. Such variations may stem from inter-study differences in TC source, bioactive composition, and dosage regimens.

UC is a chronic relapsing IBD characterized by recurrent mucosal inflammation of the colon. The sustained inflammatory response in UC has been implicated in microvascular dysfunction and endothelial barrier defects (Yin et al., 2020). DSS-induced damage to intestinal epithelial cells exacerbates colonic inflammation through upregulation of pro-inflammatory mediators, including IL-1β, IL-6, and TNF-α (Jin et al., 2020; Sanders et al., 2011). TNF-α, a pivotal driver of mucosal injury in UC (Nakase et al., 2022). Studies have shown that TNF-α can up-regulate and activate MLCK, thereby phosphorylating myosin light chain. This phosphorylation triggers the contraction of the cytoskeleton, eventually leading to changes in the position of tight junction proteins and increased intestinal permeability (Aida et al., 2020). TNF-α, IFN-γ, IL-1β, and IL-6 are pivotal pro-inflammatory cytokines, and their elevated levels significantly increase susceptibility to inflammatory pathologies associated with systemic homeostatic dysregulation. In this study, quantitative analysis of colonic inflammatory cytokines revealed that DSS administration significantly upregulated pro-inflammatory cytokine levels, including TNF-α, IFN-γ, IL-1β, and IL-6. Notably, preemptive intervention with TC and FTC could inhibit the expression of inflammatory factors. TC administration has been shown to reduce colonic expression of the pro-inflammatory cytokine IL-6 in CRC mouse models (Korish, 2014). Anti-inflammatory efficacy of TC was further validated in radiation-induced intestinal injury in C57BL/6J mice, where TC supplementation significantly lowered serum IL-1β and TNF-α levels (Chen Y. Z. et al., 2021). IL-10 and IL-4, classified as anti-inflammatory cytokines (Fung et al., 2016; Leung et al., 2016), effectively suppress inflammatory responses. In this study, TC and FTC groups significantly elevated IL-10 levels compared to DSS, with FTC demonstrating superior efficacy (Figures 3E,F). The potent anti-inflammatory effects of IL-10 are largely mediated through its ability to suppress the NF-κB signaling pathway. IL-10 inhibits the activation of IκB kinase (IKK), thereby preventing the degradation of IκB and the subsequent nuclear translocation of NF-κB, a master regulator of pro-inflammatory gene transcription (Bhattacharyya et al., 2004; Yang et al., 2025). IL-4 mainly sends signals through the JAK-STAT pathway. After binding to the receptor, IL-4 activates JAK1 and JAK3, leading to phosphorylation and dimerization of the transcription factor STAT6 (Murray, 2017). It plays a key role in tissue repair and inflammation resolution. These interventions suppressed pro-inflammatory cytokine expression while enhancing anti-inflammatory responses, thereby mitigating colonic inflammation, with FTC exhibiting enhanced therapeutic effects.

The downregulation of tight junction proteins (e.g., ZO-1, occludin, E-cadherin) facilitates intestinal pathogen and toxin infiltration across the colonic barrier, representing a key pathogenic mechanism in DSS-induced UC (Yue et al., 2019). Results demonstrated that TC and FTC groups significantly upregulated expression of colonic ZO-1, occludin, and E-cadherin compared to DSS, with FTC exhibiting superior efficacy. SCFAs, primarily composed of acetate, propionate, and butyrate, are microbial metabolites derived from the fermentation of dietary fiber. These molecules exert multifaceted roles in modulating intestinal homeostasis, immune responses, and systemic metabolism (Shin et al., 2023). SCFAs have been demonstrated to enhance intestinal barrier integrity by upregulating the expression of tight junction proteins, such as occludin and ZO-1, thereby alleviating the severity of DSS-induced UC in mice (He et al., 2023), extending to enhance the differentiation of regulatory T cells, which is a key mechanism for maintaining immune tolerance in mouse and human studies (Smith et al., 2013). In this study, the concentrations of three SCFAs in fecal were significantly reduced in DSS-induced mice compared to CK group. Prophylactic intervention with FTC and TC upregulated SCFA levels, with acetate and propionate exhibiting significant elevation relative to UC mice. Notably, butyrate exerted protective effects by upregulating tight junction proteins (e.g., ZO-1 and occludin) and suppressing NLRP3 inflammasome activation in colonic tissues (Xu et al., 2024). At the same time, butyric acid can also inhibit histone deacetylase (HDAC) in intestinal epithelial cells, thereby promoting the expression of anti-inflammatory genes (such as IL-10) and reducing the expression of pro-inflammatory cytokines such as TNF-α and IL-6 (Kibbie et al., 2021). In this study, it was also found that DSS-induced UC mice significantly down-regulated the content of butyric acid in feces, while TC and FTC groups up-regulated it, which was consistent with previous studies.

The role of intestinal flora in controlling host immune response has attracted more and more attention, and the immune system can affect the composition of intestinal flora (Mo et al., 2022). Many studies have shown that gut microbiota plays a key role in the attenuation or development of UC, and inflammation caused by UC can lead to the loss of microbial diversity, thus forming a unique microbial community composition (Edelblum et al., 2012; Monk et al., 2016; Wu et al., 2017). We observed that DSS treatment induced a significant imbalance in the gut microbiota composition of mice. Both TC and FTC partially restored the microbial community, as reflected in its diversity and structure. However, differences in their preventive effects against ulcerative colitis and their regulatory roles in gut microbiota modulation remain poorly understood. But studies have reported that TC can change the intestinal microbiota to a certain extent, while inhibiting the proliferation of certain bacteria that cause potential health problems (Wen et al., 2017). Our study showed that DSS-induced mice emerged an imbalance in the composition of the gut microbiota, and preemptive intervention with TC and FTC disrupted the DSS induced gut microbiota imbalance, and diversity and complexity of gut microbiota in FTC group were higher than DSS group, while the gut microbiota in mice of TC group was closer to DSS mice. It can be seen that in terms of flora diversity, early intervention of FTC can regulate DSS-induced intestinal flora imbalance, while the TC group cannot, which is inconsistent with the report of Wen (Wen et al., 2017). This discrepancy may be explained by differences in the specific DSS-induced colitis model, which could lead to varying degrees of dysbiosis, and/or intrinsic variations in the TC composition (e.g., originating from different breeds or diets) that ultimately affect its prebiotic efficacy. The precise reason for this difference warrants further investigation. The dominant bacteria in mice intestinal tract of the four groups were Bacteroidetes and Firmicutes (Wu et al., 2011). The relative abundance of Bacteroidetes in the mice intestinal tract of the DSS group increased, and the relative abundance of Firmicutes decreased, which was consistent with the results of DSS-induced colitis mice reported (Chen et al., 2019). Compared with the DSS group, both the TC and FTC groups exhibited an increase in the relative abundance of Firmicutes, but a decrease in Bacteroidetes and the (B/F) ratio. It is indicated that the early intervention of TC and FTC can resist the imbalance of intestinal flora in mice induced by DSS. We also found that the abundance of beneficial bacteria in the colon of DSS-induced UC in mice decreased, and the abundance of harmful bacteria increased, which was consistent with the changes of intestinal flora in UC patients (Zhang et al., 2016). This shift in the B/F ratio is associated with the amelioration of colitis, consistent with previous reports linking a lower ratio to improved intestinal health; however, its functional role in UC pathogenesis requires further mechanistic investigation. In addition, our research proved that the relative abundance of *Lachnospiraceae* NK4A136 group in the TC and FTC group was higher than that in the DSS group. Studies have shown that the *Lachnospiraceae* NK4A136 group belongs to intestinal beneficial bacteria, and higher abundance can reduce intestinal inflammation, diarrhea, and other symptoms (Wu et al., 2021) The relative abundance of Ruminococcaceae UCG-014 in the colon of mice by preemptive intervention with FTC was higher than that induced by DSS directly. Ruminococcaceae UCG-014 can produce acetate, which can inhibit the signaling pathway of proinflammatory cytokines (Yang et al., 2020). *Akkermansia* is a intestinal beneficial bacterium that can reduce the symptoms of enteritis and prevent the occurrence and development of enteritis (Herp et al., 2019). However, surprisingly, we found that the abundance of *Akkermansia* in the DSS, FTC, and TC groups was higher than that in the CK group. The low level in the CK group may reflect a state of nutritional competition within a diverse and stable gut ecosystem. In contrast, its increase upon DSS challenge could represent a compensatory host response to mucus layer damage. Notably, the elevated abundance of *Akkermansia* in the TC and FTC groups, compared to the CK group, suggests that these interventions actively facilitated the recruitment or expansion of this beneficial bacterium as part of the mucosal healing process, rather than merely reflecting a dysbiotic state. So the beneficial effects of *Akkermansia* are context-dependent and are maximized during periods of barrier disruption and repair. Through the correlation analysis of various factors, we have discovered a contradictory phenomenon: multiple strains of probiotics are significantly positively correlated with pro-inflammatory factors and butyric acid content. This finding cautions against a simplistic interpretation of their role as purely beneficial or detrimental. We propose two non-mutually exclusive explanations: (1) These taxa may be upregulated in the inflammatory environment as part of a feedback loop, potentially to regulate the immune response. (2) Their correlation with TNF-α might reflect an indirect association where both the bacteria and the cytokine increase in parallel during the active disease state, without a direct causal link. Therefore, while these genera are undoubtedly central to the colitis landscape observed here, their exact functional impact requires deeper mechanistic validation. *Ruminococcaceae* UCG-010 was positively correlated with pro-inflammatory factors and negatively correlated with anti-inflammatory factors, indicating that this strain may promote the development of UC. It has been reported that the content of pro-inflammatory factors in the intestinal tract of UC mice decreased, and the content of anti-inflammatory factors increased. There is a positive correlation between beneficial bacteria and anti-inflammatory factors, and there is a positive correlation between harmful bacteria and pro-inflammatory factors (Liu et al., 2020). The results of this study are consistent with the results of this report. In this study, the correlation analysis of intestinal flora with inflammatory factors and tight junction proteins was similar to the above results.

Although our results indicate that FTC may exert its benefits by regulating the intestinal microbiota to increase SCFAs content, thereby strengthening the intestinal barrier, the current relevant data cannot rule out other potential mechanisms, such as the direct anti-inflammatory effect of FTC components on the epithelium. Therefore, this conclusion needs to be verified by a more direct verification chain. Future work should include a series of experiments such as bacterial transplantation, direct supplementation of SCFAs to evaluate their effects in UC, and SCFAs receptor interference experiments. The confirmation of this pathway will strongly establish the status of FTC as a microbiome targeted therapeutic agent.

This study has several limitations that should be acknowledged. First, the exclusive use of a female mice, limits the generalizability of our findings to male mice. Second, although we employed a blocked randomization strategy and Tukey’s *post-hoc* test for planned comparisons, some exploratory analyses may still benefit from more stringent correction for multiple comparisons. Third, the dose of TC was selected based on preliminary studies and existing literature, and a comprehensive dose–response relationship remains to be established. Then, during the experiment, the effect of bioactive substances (such as lactoferrin, immunoglobulin) in TC on inflammation in mice was ignored, and the influence of this factor on the experiment could not be excluded. Finally, our current study is a small sample size (*n* = 3) study, and the samples only stay in mice. Future studies should include both sexes to validate the broader applicability of our findings and employ techniques such as metagenomics and bacterial culture to provide deeper mechanistic insights.

## 5 Conclusion

Early intervention with TC, particularly FTC, prevents DSS-induced colitis in mice by ameliorating histopathological damage and inflammation. This protective effect is associated with the modulation of gut microbiota, which counteracts DSS-induced dysbiosis and enhances the production of protective SCFAs. The subsequent reinforcement of colonic barrier function and the anti-inflammatory cytokine IL-10 supports the hypothesized microbiota–SCFAs–barrier axis. Future work should validate this causal chain—for instance, through fecal microbiota transplantation, germ-free models, the use of SCFAs receptor blockers, and heat-inactivated fermented TC. These findings suggest that TC and FTC may serve as potential dietary interventions for modulating gut microbiota and maintaining intestinal health; however, further clinical and mechanistic studies are required to confirm their efficacy in humans.

## Data Availability

The datasets presented in this study can be found in online repositories. The names of the repository/repositories and accession number(s) can be found at: https://www.ncbi.nlm.nih.gov/, PRJNA1260193.

## Footnotes

1
https://www.i-sanger.com/

2
www.omicstudio.cn

## Glossary

UC: Ulcerative colitis
TC: Camel milk
FTC: Fermented camel milk
DSS: Dextran sulfate sodium
DAI: Disease activity index
SCFAs: Short chain fatty acids
IBD: Inflammatory bowel disease
WHO: World Health Organization
IPA: Indole-3-propionic acid
ASD: Autism spectrum disorder
STZ: Streptozotocin
CLA: Conjugated linoleic acid
LAB: Lactic acid bacteria
CCl₄: Carbon tetrachloride
SPF: Specific pathogen-free
HE: Hematoxylin and eosin
qRT-PCR: Quantitative reverse transcription polymerase chain reaction
GC-FID: Gas chromatography-flame ionization detector
OUT: Operational taxonomic unit
TJPs: Tight junction proteins
IHC: Immunohistochemical
LEfSe: Linear discriminant analysis effect size
